# Computational Toxicology of Chloroform: Reverse Dosimetry Using Bayesian Inference, Markov Chain Monte Carlo Simulation, and Human Biomonitoring Data

**DOI:** 10.1289/ehp.11079

**Published:** 2008-04-26

**Authors:** Michael A. Lyons, Raymond S.H. Yang, Arthur N. Mayeno, Brad Reisfeld

**Affiliations:** 1 Quantitative and Computational Toxicology Group; 2 Department of Environmental and Radiological Health Sciences and; 3 Department of Chemical and Biological Engineering, Colorado State University, Fort Collins, Colorado, USA

**Keywords:** Bayesian, biomonitoring, chloroform, Markov chain Monte Carlo, MC, MCMC, Monte Carlo, PBPK, reverse dosimetry

## Abstract

**Background:**

One problem of interpreting population-based biomonitoring data is the reconstruction of corresponding external exposure in cases where no such data are available.

**Objectives:**

We demonstrate the use of a computational framework that integrates physiologically based pharmacokinetic (PBPK) modeling, Bayesian inference, and Markov chain Monte Carlo simulation to obtain a population estimate of environmental chloroform source concentrations consistent with human biomonitoring data. The biomonitoring data consist of chloroform blood concentrations measured as part of the Third National Health and Nutrition Examination Survey (NHANES III), and for which no corresponding exposure data were collected.

**Methods:**

We used a combined PBPK and shower exposure model to consider several routes and sources of exposure: ingestion of tap water, inhalation of ambient household air, and inhalation and dermal absorption while showering. We determined posterior distributions for chloroform concentration in tap water and ambient household air using U.S. Environmental Protection Agency Total Exposure Assessment Methodology (TEAM) data as prior distributions for the Bayesian analysis.

**Results:**

Posterior distributions for exposure indicate that 95% of the population represented by the NHANES III data had likely chloroform exposures ≤ 67 μg/L in tap water and ≤ 0.02 μg/L in ambient household air.

**Conclusions:**

Our results demonstrate the application of computer simulation to aid in the interpretation of human biomonitoring data in the context of the exposure–health evaluation–risk assessment continuum. These results should be considered as a demonstration of the method and can be improved with the addition of more detailed data.

To understand the effects on public health of exposure to environmental chemicals requires establishing relationships among events along an exposure–health evaluation–risk assessment continuum (National Research Council 2006). Biomonitoring data, such as chemical concentrations in tissues and fluids, are a measure of internal exposure and represent one event along the continuum to be linked with external exposure and biologically effective dose. We focus here on the relationship between internal and external exposure, with external exposure being a measure of environmental chemical concentration in contact with the body. Often, however, biomonitoring data are reported without corresponding external exposure data, leaving the relationship between internal and external exposure as one to be determined; establishing this relationship involves the reconstruction of past external exposure or dose, from biomonitoring data collected at some later time. Such exposure reconstruction can be addressed at both the individual and the population level. A procedure for determining an estimate of external exposure consistent with biomonitoring data measured in a population has been termed “exposure reconstruction” or “reverse dosimetry.”

A population-based estimate of exposure should account for the intrinsic heterogeneity (variability) in the population, both in the modeling of the disposition of the chemical in the body, and in the description of the exposure conditions. Additionally, the biomonitoring information itself, considered as a whole, should reflect the variability in the population from which it arises.

Tan et al. (2006) incorporated variability into the reverse dosimetry of chloroform using a combined physiologically based pharmacokinetic (PBPK) and shower exposure model, with external exposure calculated using an exposure conversion factor (ECF) distribution. The ECF distribution was obtained by inverting the output of a Monte Carlo (MC) simulation for chloroform concentration in blood using, as input, a preselected reference value for chloroform concentration in tap water. The product of the ECF distribution with an observed blood concentration provides a distribution of tap water concentrations corresponding to that blood level.

Although the ECF distribution provides a population estimate of exposure, its accuracy is limited to the case in which tissue dose is linearly related to external exposure. In this article, we reconsider the work of Tan et al. (2006) with an approach to reverse dosimetry using Bayesian inference in place of the ECF distribution.

In addition to the work of Tan et al. (2006), exposure reconstruction for chloroform using PBPK modeling has appeared in Georgopoulos et al. (1994) and Roy et al. (1996) in the form of a maximum likelihood calculation. In this case, the biological model parameters remained as fixed values representing an average or reference individual and did not account for population variability. The report by Tan et al. (2007) included a calculation of chloroform tap water concentration using Bayes’ theorem, but with the prior distribution taken as unity, which reduced Bayes’ theorem to a maximum likelihood calculation.

Previous work using Bayesian methods for exposure reconstruction for other chemicals has appeared in Miller et al. (2002), Sohn et al. (2004), and Allen et al. (2007). The work of Miller et al. (2002) described a general procedure for individual dose reconstruction using Bayesian inference and Markov chain Monte Carlo (MCMC) simulation. In this case, the model for chemical disposition was a traditional compartment-based kinetic model for plutonium-239, which was applied to individual exposure reconstruction for ^239^Pu from urine measurements. Although the model used was not a PBPK model and did not address population-based measurements and variability, it served as the motivating example in formulating the details of the method we present in this article.

Sohn et al. (2004) used a PBPK model and Bayesian inference to reconstruct exposure to trichloroethylene from detailed concentration–time data for eight individuals. They obtained a population estimate by treating the individuals as a random sample from a larger population without the use of a hierarchical population model or the use of MCMC simulation. They evaluated Bayes’ theorem directly by using MC simulation to build up a library of terms for the likelihood and prior incorporating distributions for PBPK model parameters to account for population variability.

Allen et al. (2007) recently reconstructed exposure to methylmercury (MeHg) in women of childbearing age and pregnant women, using a method similar to that presented here. Their application involved two stages of Bayesian updating to recalibrate the PBPK model parameters along with oral absorption of MeHg for the subpopulation of interest. Both the method of Allen et al. (2007) and the method presented here are based on the work of Gelman et al. (1996), which was presented as a general method of parameter estimation in PBPK models. This method originally was applied to PBPK model calibration, and examples and reviews can be found in a number of articles (Bernillon and Bois, 2000; Covington et al. 2007; Hack et al. 2006; Jonsson 2001).

We view reverse dosimetry as a type of PBPK model calibration problem, which allows us to use established methods and tools to aid in the interpretation of population-based biomonitoring data.

## Reverse dosimetry

The fundamental problem underlying reverse dosimetry is to relate a measured internal dose, or tissue concentration, *C_T_*, to an unmeasured external exposure or dose, *C_D_*, given a deterministic model *f* (we consider *f* to be minimally a PBPK model). The usual mode of operation for *f* is to solve the “forward problem” of determining the tissue concentration given a known external dose: *C_T_* = *f* (*C_D_*). If our model is such that an appropriate inverse *f* ^−1^ can be found, then the reverse problem can be solved as *C_D_* = *f* ^−1^(*C_T_*). Typically, however, *f* is such that an inverse either does not exist or may not be unique, or may be unstable, meaning that a small change in the data may lead to a large change in output of the inverse function; that is, the reverse problem is usually “ill-posed” (Hadamard 1902). Additional complications arise when considering the population-based nature of the biomonitoring data where population variability becomes a significant factor that needs to be incorporated into the solution for *C_D_*. Also, biomonitoring data represent accumulation of chemicals in the body from all possible sources and routes of exposure, and we may need to account for multiple simultaneous independent inputs into the model.

## Bayesian inference

A Bayesian approach determines *C_D_* as a probability distribution rather than a single value, the starting point being the treatment of all observables and parameters of interest as random variables. The external dose *C_D_* is assigned a “prior” probability distribution representing knowledge about *C_D_* before consideration of the data *C_T_*. The prior distribution is updated via Bayes’ theorem, into a “posterior” probability distribution for *C_D_* conditioned on the data *C_T_*. Bayes’ theorem can be written as


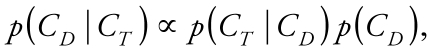


where *p*(*C_D_*) is the prior, *p*(*C_T_* |*C_D_*) is the likelihood, and *p*(*C_D_* |*C_T_*) is the posterior. The likelihood is the conditional distribution *p*(*C_T_* |*C_D_*) viewed as a function of *C_D_* and whose functional form is based on the specification of a measurement model that describes the difference between observation and model prediction in terms of an error.

A significant aspect of PBPK models is that all of the parameters have a physical or biological interpretation: they are not arbitrary. We can use knowledge regarding possible ranges, central values, and measures of dispersion, as well as specific data from separate studies, to define informative prior distributions.

The product of the prior and likelihood gives (up to a normalization constant) the posterior distribution containing all information regarding the parameter *C_D_* consistent with the data and prior information. The posterior distribution is the solution of the reverse problem, and all further inferences regarding *C_D_* is made from it in terms of expectation values of functions of *C_D_*.

For most cases of practical interest, the normalized posterior distribution is evaluated via numerical simulation. MCMC simulation is the standard method used for Bayesian analysis (Gelman and Rubin 1996; Gilks et al. 1996). MCMC simulation refers to a class of iterative simulations in which the random variables of interest are drawn from a sequence, or chain, of distributions that eventually converge to a stable posterior distribution. These chains can be determined by rejection sampling algorithms where a random draw is accepted or rejected based on a simple probabilistic rule (e.g., the Metropolis-Hastings algorithm; see Gilks et al. 1996). Convergence can be assessed by running multiple chains and comparing the variance within and between the sequences via a “potential scale reduction” factor R̂ (Gelman et al. 2004). R̂ is such that lim_n→∝_R̂ = 1, where *n* is the number of iterations. Gelman et al. (2004) recommend continuing iterations until R̂ < 1.1 for each parameter of interest. Once convergence is obtained, the multiple chains can be aggregated and considered to be a sample from a discrete approximation to the posterior distribution. The expectation value *E*[*h*(*C_D_* )] of an arbitrary function *h*(*C_D_* ) can be estimated by drawing {*C_Dk,_* *k =* 1, . . . , *N* } from the posterior and calculating the following:


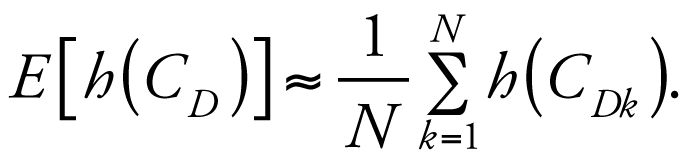


For example, we can estimate the expected value for *C_D_* as the sample mean of the posterior distribution. Note also that *C_D_* can consist of multiple components, the posterior being a joint distribution from which we can calculate marginal distributions for each component.

The above discussion describes the basic elements of a Bayesian analysis that would apply to an estimate of external dose for an individual based on data collected for that individual. The reverse dosimetry problem, however, is a problem of statistical inference: we wish to determine an estimate of exposure for the general population based on biomonitoring data collected from a representative sample of that population. We can address this statistical aspect of the problem by combining Bayesian analysis with a population model.

## Materials and Methods

We applied the Bayesian population analysis of Gelman et al. (1996) to the problem of reverse dosimetry for chloroform to obtain population estimates of chloroform concentrations in tap water and ambient household air under residential exposure conditions. We viewed reverse dosimetry as a type of model calibration problem where, using an otherwise calibrated model, we determined unmeasured exposure parameters based on the measured biomonitoring data. The basic elements of the analysis include a PBPK + shower model, prior chloroform concentration measurements in tap water and ambient air from the U.S. Environmental Protection Agency (EPA) Total Exposure Assessment Methodology (TEAM) study (Wallace 1997), and biomonitoring data in the form of chloroform concentrations in blood measured as part of the Third National Health and Nutrition Examination Survey (NHANES III) (Centers for Disease Control and Prevention 1996). With some noted exceptions, we use the PBPK + shower model, parameter distributions, definition of exposure, and experimental data provided in Tan et al. (2006, 2007); however, these elements have a different rationale and purpose in the context of the Bayesian population framework presented here, than that of the ECF distribution or likelihood-based methods.

The TEAM study data we used here were collected during 1981–1984 from a different population than that used for the NHANES III data, which were gathered during 1988–1992. No exposure data were collected corresponding to the NHANES III biomonitoring data, and the objective here is to determine an estimate for such corresponding exposure.

The reverse dosimetry problem for chloroform is to relate a sample of chloroform blood concentrations, *C_V_*, to an unmeasured population distribution of environmental chloroform source concentrations, *C_S_*, given the deterministic model *f : C_S_* → *C_V_*. The deterministic model *f* = *f* (*t*, φ, *C_S_*) is the PBPK + shower model and represents the solution to a set of differential equations derived from biological and physical principles considered to be common to all members of the population; it is a function of time *t* and a set of parameters whose values distinguish the various individuals. We divided the parameters into those that are to be updated in the analysis, that is, the unmeasured source concentrations *C_S_* = (*C_W_*, *C_A_*), and those that are to remain fixed, where *C_W_* and *C_A_* are the concentrations in water and air, respectively. We designate the fixed parameters by φ, which can be single-point values or fixed distributions representing pharmacokinetic, shower model, and other exposure parameters such as drinking water intake and shower duration.

**Figure 1:**
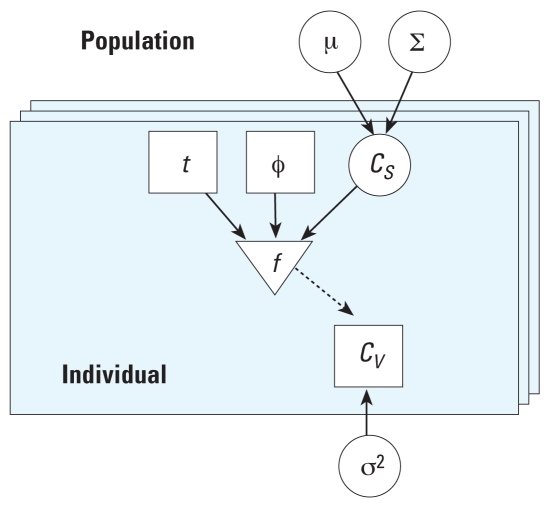
Graphic model (adapted from Bernillon and Bois 2000). Circles represent unknown quantities to be updated via Bayes’ theorem: population mean (μ) and variance (∑), concentrations (*C_S_*), and error (σ^2^). Squares represent the known quantities of time (*t*), PBPK model and exposure parameters (φ), and measured blood concentrations (*C_V_*). The triangle represents the deterministic model (*f* ). The solid arrows represent conditional dependence, and the dashed arrow represents a deterministic link. Individuals are represented by the layered boxes, and are considered to be a subset of the population.

Population variability is described by considering individual values for *C_S_* to arise independently from a population distribution parameterized by a population mean μ and a population variance ∑. The introduction of population parameters induces a hierarchical structure among the model parameters that, along with the specification of the deterministic model quantities and error, defines the population model. The population model, specifying the conditional dependencies among the population and individual parameters and the link through the deterministic model to the data and error, can be summarized as a graphical model (Figure 1). Here, blood and source concentrations are related at the individual level through the deterministic model, with source and error parameter values for each individual arising independently from population-level distributions.

The Bayesian analysis proceeds as described above, but with the additional structure among the parameters in the population model incorporated into the terms in Bayes’ theorem. Writing the joint prior probability distribution as *p*(μ, ∑, *C_S_*, σ^2^), we use the conditional dependencies encoded in the graphical model to obtain *p*(μ, ∑, *C_S_*, σ^2^) = *p*(μ) *p*(∑) *p*(*C_S_* | μ, ∑) *p*(σ^2^). Similarly, the likelihood is *p*(*C_V_* |μ, ∑, *C_S_*, σ^2^) = *p*(*C_V_* |*C_S_*,σ^2^). Bayes’ theorem then takes the form


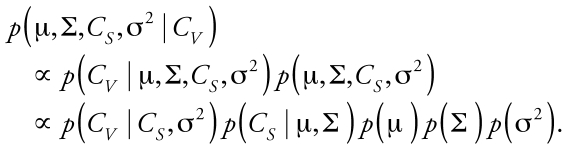


Once we specified the prior distributions for μ, ∑, and σ^2^, the next step was to calculate the posterior distribution conditioned on the observed data and to calculate the statistical quantities for the parameters of interest.

**Figure 2:**
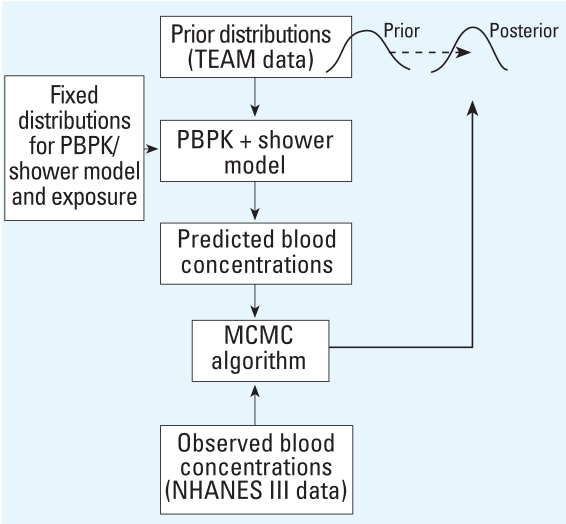
Basic elements for reverse dosimetry of chloroform using Bayesian analysis.

Figure 2 illustrates the relationships among the basic elements used in the Bayesian analysis. Random draws from the prior distributions for water and air concentrations, and from fixed distributions for pharmacokinetic, shower model, and other exposure parameters, define individual parameter sets from which we calculated model predictions for chloroform blood concentrations. We compared predicted blood concentrations with the observed concentrations and accepted the sampled values for water and air with probability defined in the MCMC algorithm. Using the output parameters of one iteration as the input for the next, we repeated the procedure until the parameter distributions for water and air became stable, and then transformed the prior distribution to the posterior distribution.

The analysis of the reverse dosimetry problem consists of the following steps:

- Specification of the probability model: specification of the joint probability distribution incorporating the PBPK + shower model, hierarchical population model, measurement model, and the specification of prior parameter distributions
- Bayesian inference: calculation of the posterior distribution conditioned on the observed biomonitoring data using MCMC simulation and calculation of expected values for exposure
- Evaluation of the results: comparison of prior and posterior distributions of exposure using MC simulation to generate model predictions for the observed biomonitoring data, evaluation of parameter independence, and comparison with previously obtained results.

We performed all model simulations using MCSim, version 5.1 beta (Bois and Maszle 1997), compiled and run on an Intel Pentium 4 CPU (2.80 GHz) with Linux kernel 2.6.17–12. MCSim uses LSODES (Hindmarsh 1983) as the differential equation solver, and Metropolis-Hastings (Hastings 1970; Metropolis et al. 1953) sampling for MCMC simulation.

### Probability model

We placed the PBPK + shower, measurement, and population models into a probability context through the specification of distributions for the likelihood and priors. We then combined these into an expression for the posterior distribution for Bayesian analysis.

### PBPK + shower model

**Figure 3:**
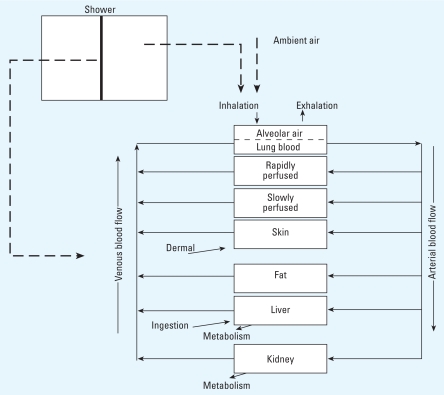
Schematic of PBPK + shower model for chloroform (Tan et al. 2006).

The PBPK + shower exposure model consists of a PBPK model for chloroform (Corley et al. 2000) combined with a mass transfer model for chloroform volatilized from shower water (Weisel et al. 1999). We consider the model validated for the forward problem under controlled experimental conditions; that is, the model accurately predicts measured concentration–time profiles for a known external dose. Figure 3 illustrates this model.

The shower model consists of a shower stall in which chloroform is volatilized from a plug flow stream of tap water into well-mixed shower stall air. Model parameters include shower water flow rate, shower stall volume, and a chloroform mass transfer coefficient accounting for details of the shower system that were not explicitly modeled (e.g., shower head design).

The PBPK model consists of seven compartments with chloroform exposure specified as inhalation, dermal, and ingestion. We consider inhalation exposure to be from chloroform in ambient household air and from chloroform volatilized from shower water during showering. Inhalation exposure is indicated in the gas exchange compartment, which we consider to be under equilibrium and steady-state conditions. We define dermal exposure as a net flux of chloroform into the skin via passive diffusion from direct contact with water while showering. Ingestion is via drinking water, which we indicate as absorption directly into the liver. Elimination of chloroform is through exhalation from the gas exchange compartment and metabolism in the liver and kidney compartments.

In order to maintain physiologic constraints during MC and MCMC simulations, we made the following modifications to the PBPK model (see, e.g., Marino et al. 2006): *a*) correlating cardiac output with alveolar ventilation rate through the ventilation perfusion ratio (ventilation rate/cardiac output), *b*) constraining fractional blood flows to sum to unity by dividing the fractional blood flow to each tissue by the sum of fractional blood flows to all tissues, and *c*) constraining fractional tissue volumes to sum to 0.91, by multiplying each fractional tissue volume by 0.91 and dividing by the total fractional tissue volume. We chose value 0.91 to match the total fractional tissue volume used previously (Tan et al. 2006).

### Pharmacokinetic parameters

With the exception of body weight and the ventilation perfusion ratio, all pharmacokinetic parameter values and distributions are those given in Tan et al. (2006). We describe body weight with a normal distribution with mean of 70 kg (Brown et al. 1997) and coefficient of variation of 30%. We took the ventilation perfusion ratio as lognormal with a mean of 1.45 and a coefficient of variation of 18%, calculated from the cardiac output and alveolar ventilation data given in Tan et al. (2006).

### Exposure parameters

Consistent with Tan et al. (2006), we defined exposure in terms of the following seven parameters: *a*) chloroform concentration in tap water, *b*) chloroform concentration in ambient household air, *c*) shower duration, *d*) shower water flow rate, *e*) shower stall dimensions, *f* ) chloroform mass transfer coefficient, and *g*) daily drinking water intake.

Distributions for several of the exposure parameters were provided by Tan et al. (2006), who used them to generate distributions for MC simulation equivalent to a linear interpolation between data points. Here, we fitted smooth normal or lognormal distributions to the percentile data (Table 1). We obtained all curve fits to percentile data using Gnuplot, version 4.0 (Gnuplot 2007), which uses a nonlinear least-squares algorithm (Marquardt-Levenberg) to determine a best fit.

We truncated these parameter distributions to include 95% of the distribution (mean ± 1.96 SD for a normal distribution) to avoid sampling from implausible values and to be consistent with the truncations used previously (Tan et al. 2006).

The notation θ ~ *N*(*M*, *S* ^2^) indicates the parameter θ is distributed normally with mean *M* and variance *S* ^2^. For data that are lognormally distributed with sample mean *M* and variance *S* ^2^, the notation


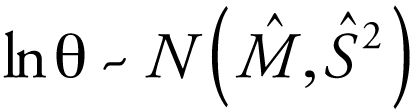


indicates the log-transformed quantity is normally distributed with the following mean and variance:


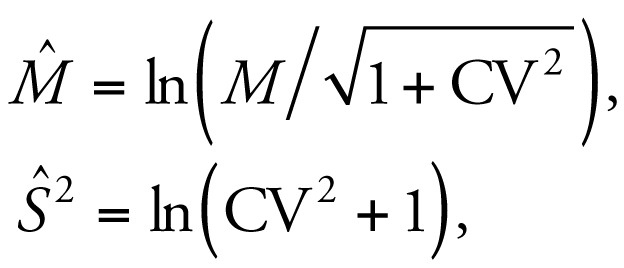


where the coefficient of variation (CV) = *S* /*M*. We also used geometric mean GM = exp(M̂ ) and geometric standard deviation GSD = exp(Ŝ) to characterize the central value and dispersion for the lognormally distributed quantities.

**Figure 4:**
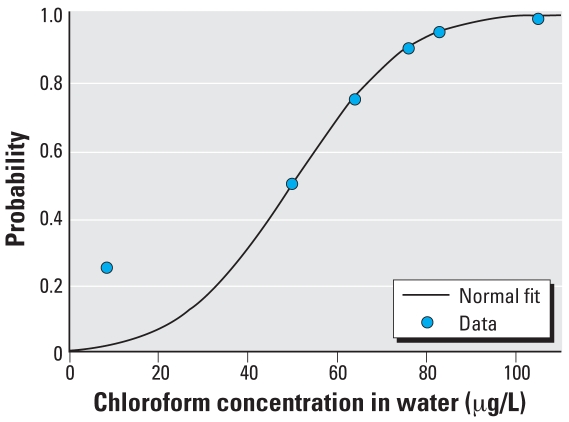
Prior distribution function for chloroform concentration in tap water; curve-fit and percentile data.

We found no significant deviations of the curve fits from the percentile data, with the exception of that for chloroform concentration in tap water, which underestimates the 25th percentile point (Figure 4). The data we used for chloroform concentration in tap water (Wallace 1997) consisted of three samples from Bayonnne–Elizabeth, New Jersey, and three samples from Los Angeles and Antioch–Pittsburg, California. The 25th percentile point came only from the California samples that had concentration values approximately half that of the New Jersey measurements for the other percentiles. Also, in the context of the reverse dosimetry problem addressed here, we considered the parameters *C_W_* and *C_A_* to be unmeasured. We used the distributions for these terms to define prior distributions, which we will update based on the measured biomonitoring data. Because we have a good fit to the median and upper percentile values, we maintain the fitted curve as a reasonable prior approximation for tap water concentrations.

### Population and measurement models

Figure 2 graphically depicts the population model describing the relationships among model quantities, parameters, and observables. The source concentration *C_S_* consists of two independent components corresponding to tap water and ambient air concentrations *C_S_* = (*C_W_*, *C_A_*). The population mean and variance terms are then μ = (μ*_W_*, μ*_A_*) and ∑ = (∑*_W_*, ∑*_A_*). The variance σ^2^ consists of only a single component corresponding to error in chloroform blood concentration measurements. The population model specifies the relationship among parameters before consideration of the data; it may be that conditioning on the data induces a correlation among components initially specified as independent.

For the measurement model, we considered *I* individuals, from each of whom we simulated a single chloroform blood concentration *C_Vi_* at time *t_i_*, *i* = 1, . . . , *I*. We used the lognormal measurement model (Bernillon and Bois 2000), ln(*C_Vi_*) = *f* (*t_i_*, φ*_i_*, *C_Wi_*, *C_Ai_*) + ɛ*_i_*, *i* = 1, . . . , *I*, where *f* is the PBPK + shower model, φ*_i_* are the fixed pharmacokinetic, shower model, and exposure parameters for each individual, and the error terms ɛ*_i_* ~ *N*(0, σ^2^). The likelihood then takes the form


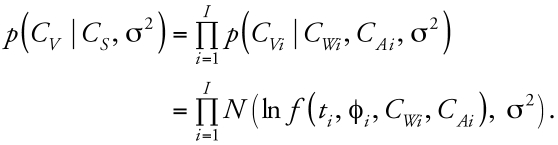


### Prior distributions

We assigned prior distributions for each component of the population mean μ = μ(*M*, *S* ^2^) based on the best estimate of the mean *M* and variance *S* ^2^ for the parameter of interest; that is, we interpreted a prior estimate of the mean and variance of the parameter as a prior distribution of means for that parameter. From Table 1, we have the prior population mean distribution for chloroform concentration in tap water, μ*_W_* ~ *N*(50, (20)^2^), truncated to include the interval (10.8, 89.2). We used the log-transformed distribution for ambient air concentrations, taking the population mean distribution as ln(μ*_A_*) ~ *N*(−5.68, (1.32)^2^) truncated to include the interval (−8.27, −3.09). The truncations are such as to include 95% of the distributions, with units defined as in Table 1.

The prior distributions for the population variances ∑ are described with an inverse gamma (Inv-γ) distribution (Carlin and Louis 2000), ∑ ~ Inv-γ(α, β), where α > 0 is the shape parameter and β > 0 is the scale parameter; the mean and variance can be expressed, respectively, as


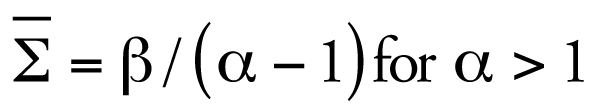


and


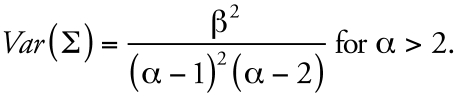


We set the prior values for α and β by setting the mean and standard deviation of ∑ equal to the variance of that population mean distribution defined by the expected value for μ. This gives α = 3, and for μ ~ *N*(*M*, *S* ^2^), we have β = (α – 1) × ∑̄ = 2*S* ^2^.

For lognormally distributed μ, with lnμ ~ *N*(M̂, Ŝ^2^), β = 2Ŝ^2^. From Table 1 then, ∑*_W_* ~ Inv-γ(3, 800) and ∑*_A_* ~ Inv-γ(3, 3.48).

A standard prior for the error distribution, σ^2^, is a noninformative log-uniform distribution, which we take over the interval [0.001, 100] (pg/mL)^2^ in natural space.

#### Bayesian inference

We calculated the posterior distribution *p*(μ, ∑, *C_S_*, σ^2^ |*C_V_*) using MCMC simulation conditioned on the observed biomonitoring data. We interpreted the expected values of the posterior population mean and variance distributions as the updated mean and variance parameters for the source concentration distributions as *C_S_* = *C_S_* μ̄, ∑̄) where μ̄ = *E*(μ) and ∑̄ = *E*(∑).

### Biomonitoring data

The biomonitoring data consist of concentrations of chloroform in blood measured as part of NHANES III, with blood sample collection times occurring between 0800 hours and 2300 hours. The blood concentrations were reported in percentile form; to obtain individual data for MCMC simulation, we first fitted the percentile data to a lognormal distribution ln(*C_V_*) ~ *N*(3.12, (0.944)^2^), where *C_V_* denotes chloroform blood concentration (picograms per milliliter).

We truncated the distribution to include 95% of the values and generated individual chloroform blood concentrations by random draws from this truncated distribution to generate data corresponding to *I* = 80 individual measurements. We chose the limit of 80 due to computational time considerations (simulations ran about 11 hr each). We distributed the sampled blood concentrations uniformly over each hour in the sampling time interval: five random data points per hour, for each hour in the interval 0800–2300 hours.

### Exposure regimen

We assumed continuous water intake at a constant rate from 0600 hours to 2200 hours, and a single shower start time of 1010 hours. We obtained the shower start time as the 50th percentile (median) of the shower start time distribution used by Tan et al. (2006). Our regimen was simplified from that of Tan et al. (2006), who used a pulsed water intake and a distribution for shower start time. The influence of the shower start time, in the context of the problem at hand, is to provide a time point that, combined with shower duration, defines an upper or lower bound to the time interval between shower exposure and sampling time. Beyond the time interval of 0800–2300 hours, no data are available regarding blood sampling time and exposure; we used the distribution of chloroform blood concentrations throughout the sampling interval to account for variation between exposure and blood sampling time. We considered inhalation of chloroform in ambient household air to be constant.

### MCMC simulation

The MCMC simulations consisted of three independent chains of 10,000 iterations. We discarded the first 5,000 iterations and assessed convergence for each of the parameters of interest using the potential scale reduction factor of Gelman et al. (2004), with R̂ < 1.1 as criteria for convergence. We then aggregated the independent chains for each parameter and considered them to be a sample from the posterior distribution.

#### Evaluation of results

##### MC simulation

We performed MC simulations of chloroform blood concentrations using both the prior and posterior distributions for chloroform tap water and ambient air concentrations along with the distributions for the PBPK + shower model and exposure parameters. We ran the MC simulations (10,000 iterations) corresponding to each hour in the interval 0800–2300 hours. We aggregated the simulations for each hour into a single distribution function and compared it with the observed blood concentrations from the NHANES III data.

### Posterior correlation of parameters

We checked the assumption of independence between tap water and ambient air concentrations through the posterior correlations that might have arisen following conditioning on the data. For each individual *i*, we have in the aggregated posterior distribution 15,000 pairs of individual tap water and ambient air concentrations (*C_Wi_*, ln(*C_A_*)*_i_*). We calculated the individual sample correlations *r_i_* = corr(*C_Wi_*, ln(*C_A_*)*_i_*), *i* = 1, . . . , *I*, and estimated the population correlation as the mean of all the *r_i_*.

### Comparison with previously obtained results

We compared the posterior chloroform tap water distribution with the results using the ECF distribution reported by Tan et al. (2006). We generated a distribution of chloroform blood concentrations as 10,000 random draws from the distribution defined by the curve fit for *C_V_*. The product of this distribution and the ECF distribution yields a distribution for chloroform concentrations in tap water.

## Results

### MCMC simulations

**Figure 5:**
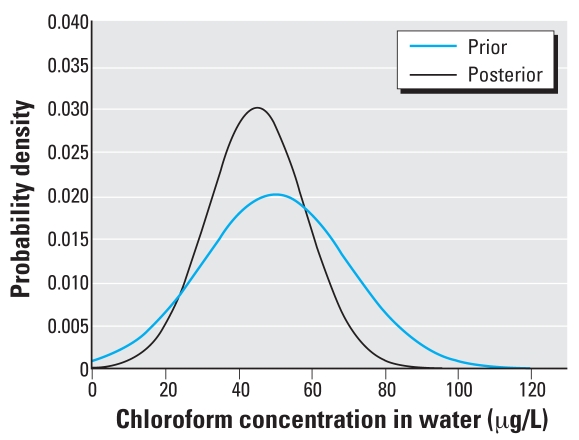
Probability density functions for prior and posterior chloroform concentrations in tap water.

**Figure 6:**
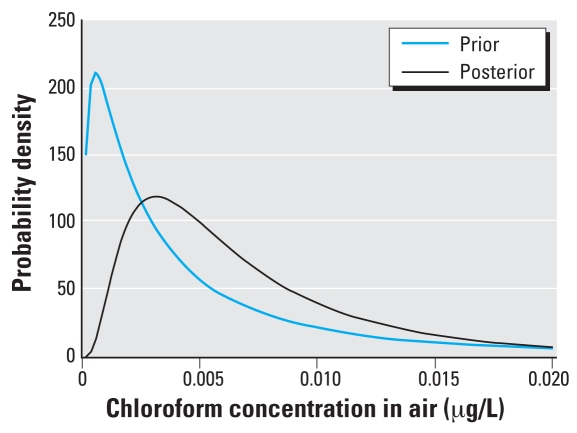
Probability density functions for prior and posterior chloroform concentrations in air.

The MCMC simulations converged to R̂ < 1.02 for each of the population mean and variance parameters. The posterior means and variances, along with the prior values, are noted in Table 2, with the probability density functions plotted in Figures 5 and 6.

The posterior distributions represent likely distributions for chloroform concentrations in tap water and ambient air consistent with the biomonitoring data, as well as the assumptions and constraints imposed by the model, exposure regimen, and prior distributions. The posterior distribution for tap water concentrations shows a decrease in the median and variance compared with the TEAM data (Wallace 1997), and the posterior distribution for ambient air concentrations shows an increase in the geometric mean and decrease in the geometric SD. A proper assessment of these results requires a direct comparison of the posterior distributions with exposure data that would correspond to the NHANES III blood concentrations, and no such data are available at this time. We also evaluated the results by comparison of model predictions for blood concentrations using the posterior distributions for exposure.

### MC simulations

**Figure 7:**
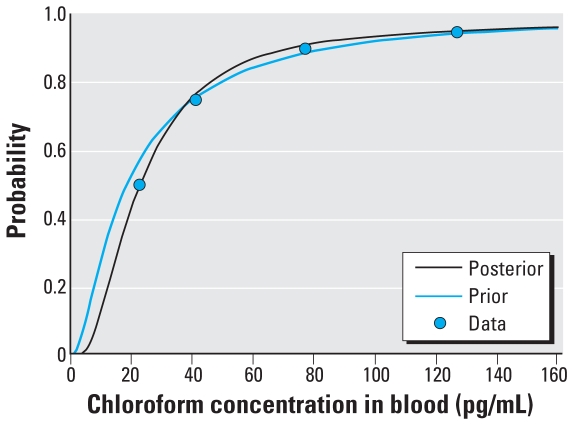
Measured and predicted concentrations of chloroform in blood using prior and posterior distributions for chloroform in tap water and ambient household air.

Table 3 and Figure 7 show the results of the MC simulations for chloroform blood concentrations using the prior and posterior concentrations for chloroform in tap water and ambient household air. The results for the prior distribution agree closely with the results reported previously (Tan et al. 2006) for the case where chloroform concentration in air is independent of that in water, indicating that the simplifications in exposure regimen used here had little effect on blood concentration compared with the more detailed regimen. The posterior values for tap water and ambient air concentrations provide a better match to the observed values than do the priors, particularly for the median blood concentration. Comparing the prior and posterior curve fits in terms of the residual sum of squares (RSS), we have RSS_prior_ = 201 (pg/mL)^2^ and RSS_posterior_ = 46(pg/mL)^2^.

### Correlation between ambient air and tap water concentrations

The average individual sample correlation between chloroform tap water and ambient air concentrations from the posterior distribution was *r*^−^ = –0.05 with SE = 0.004. We interpret this as indicating little, if any, correlation between chloroform tap water and ambient air concentrations induced by conditioning on the NHANES III data. The lack of correlation between air and water concentrations suggests that air concentration levels are more the result of other factors, such as use of cleaning products, and nonlinear mixing effects involving ventilation, use of dishwashers and washing machines, or other modes of chloroform source concentrations.

### Comparison with results from the ECF distribution approach

Table 4 shows the percentile values for chloroform concentrations in tap water from the posterior distribution in Table 2 and also as the product of measured blood concentrations and the ECF distribution.

The ECF distribution gives very high values for the upper percentiles that are not present in the results for the Bayesian approach. The results of the Bayesian analysis are consistent with the TEAM data and provide exposure distributions that lead to close agreement between model predictions of chloroform blood concentration and the observed bio-monitoring data.

Ambient air concentration was provided by Tan et al. (2006) through the relationship *C_A_* (ppm) = 0.0179 × *C_W_* (mg/L); they reported no results corresponding to a posterior distribution for the case in which these quantities are independent.

## Conclusions

In this article we presented a method for interpreting biomonitoring data in the context of the exposure–health evaluation–risk assessment continuum. The Bayesian analysis we used here relates population-based measurements of chloroform blood concentrations to chloroform exposure in terms of tap water and ambient household air concentrations given as the posterior distributions in Table 2. This places biomonitoring information in a health-based context by relating it to exposure-based quantities such as maximum contaminant level (MCL) and reference dose (RfD). With the understanding that the method we used here has not been subjected to comparison with experimental data for exposure, and that the numbers presented are for demonstration of the method, we note from Table 4 that 95% of the population represented by the NHANES III data was likely to be exposed to ≤ 67 ppb chloroform in water, which can be compared with the MCL for trihalomethanes of 100 ppb (U.S. EPA 2007). Using distributions for the posterior concentration of chloroform in tap water (*C_W_* ), daily water intake (*W_I_* ), and body weight (BW), we drew 10,000 samples from the product *C_W_W_I_* /BW to obtain a daily chloroform intake from drinking water (Table 5). The RfD for chloroform is 0.01 mg/kg/day (U.S. EPA 2001). The 95th percentile for the posterior distribution for chloroform concentration in ambient air is 0.02 μg/L. The U.S. EPA currently does not have an established inhalation reference concentration for chloroform (U.S. EPA 2001).

The accuracy of the results is limited by the approximate nature of the model, the assumptions regarding exposure, and the quality of the experimental data. In particular, the prior distribution from the TEAM data did not correspond to the same population as that of the NHANES III data, and although it provided an informative prior, data from the same locations and time frame as the NHANES III data, even if not corresponding to the individuals in that study, would likely improve the accuracy of the results. Although the method presented here is intended to be a tool to reconstruct exposure from biomonitoring data where no corresponding exposure data are available, comparison of the results with such data would greatly assist in assessing the accuracy of the method, and such results could be incorporated as prior distributions for additional chloroform dose reconstructions.

## Correction

In the abstract of the original manuscript published online, the units for chloroform exposures in tap water were presented as milligrams per liter instead of micrograms per liter. They have been corrected here.

## Figures and Tables

**Table 1:** Exposure/source distributions fit to percentile data from Tan et al. (2006).

Parameter	Distribution
*Q_W_*: Shower water flow rate (L/hr)	Lognormal ln(*Q_w_*) ~ *N* (6.132,(0.40)^2^)
*W_I_*: Water Intake (L/day)	Lognormal ln(*W_I_*) ~ *N* (−0.469,(0.872)^2^)
Δ*_SH_*: Shower duration (hr)	Lognormal ln(Δ*_SH_*) ~ *N* (−1.43,(0.58)^2^)
*C_W_*: Tap water concentration (μg/L)	Normal *C_w_* ~ *N* (50,(20)^2^)
*C_A_*: Ambient air concentration (μg/L)	Lognormal ln(*C_A_*) ~ *N* (−5.68,(1.32)^2^)

**Table 2:** Prior and posterior tap water and ambient air concentrations (geometric mean and geometric SD for *C_A_*).

Parameter	Distribution	Prior mean	SD	Posterior mean	SD
*C_W_* (μg/L)	Normal	50	20	44.9	13.3
*C_A_* (μg/L)	Lognormal	3.4 × 10^−3^	3.7	5.8 × 10^−3^	2.2

**Table 3:** Measured and predicted chloroform concentrations in blood (pg/mL).

	Percentile						
	5th	10th	25th	50th	75th	90th	95th
Measured chloroform blood concentrations [NHANES III data (pg/mL)]	—	—	—	23	41	77	127
Predicted chloroform blood concentrations
Using prior distributions for tap water and ambient air [blood (pg/mL)]	3.7	5.3	9.3	19	41	85	138
Using posterior distributions for tap water and ambient air [blood (pg/mL)]	7.3	9.3	14.0	23	40	71	124

—, not available.

**Table 4:** Comparison of chloroform concentrations using Bayesian analysis with that calculated using the ECF distribution of Tan et al. (2006, 2007).

	Percentile						
	5th	10th	25th	50th	75th	90th	95th
*C_W_* (μg/L) (posterior)	23	28	36	45	54	62	67
*C_W_* = *C_V_* × ECF (μg/L)	6	10	21	48	102	195	275

**Table 5:** Estimated chloroform intake from drinking water (mg/kg/day).

Percentile	Chloroform intake (mg/kg/day)
5th	9.9 × 10^−5^
10th	1.3 × 10^−4^
25th	2.1 × 10^−4^
50th	4.0 × 10^−4^
75th	7.4 × 10^−4^
90th	1.2 × 10^−3^
95th	1.6 × 10^−3^
