# Correction: Computational Toxicology of Chloroform: Reverse Dosimetry Using Bayesian Inference, Markov Chain Monte Carlo Simulation, and Human Biomonitoring Data

**DOI:** 10.1289/ehp.11079-erratum

**Published:** 2008-04-26

In the abstract of the original manuscript published online, the units for chloroform exposures in tap water were presented as milligrams per liter instead of micrograms per liter. They have been corrected here.

